# Efficacy and Safety of Artificial Tears Containing Lipidure and Hypromellose for the Treatment of Moderate Dry Eye Disease in Contact Lens Wearers

**DOI:** 10.3390/medicina60020287

**Published:** 2024-02-08

**Authors:** Caterina Gagliano, Marco Zeppieri, Antonio Longo, Giovanni Rubegni, Roberta Amato, Roberta Foti, Francesco Cappellani, Massimiliano Cocuzza, Federico Visalli, Ludovica Cannizzaro, Alessandro Avitabile, Giuseppe Gagliano, Lucia Lapenna, Fabiana D’Esposito

**Affiliations:** 1 Faculty of Medicine and Surgery, University of Enna “Kore”, Piazza dell’Università, 94100 Enna, Italy; caterina_gagliano@hotmail.com; 2 Eye Clinic, Catania University, San Marco Hospital, Viale Carlo Azeglio Ciampi, 95121 Catania, Italy; 3 Department of Ophthalmology, University Hospital of Udine, 33100 Udine, Italy; 4 Faculty of Medicine, University of Catania, Piazza Università, 95123 Catania, Italyludocann@gmail.com (L.C.); alessandro.avitabile2001@gmail.com (A.A.);; 5 Ophthalmology Unit, University of Siena, 53100 Siena, Italy; 6 Willis Eye Hospital, Thomas Jefferson University, Philadelphia, PA 19107, USA; 7 Department of Ophthalmology, Di Venere’s Hospital of Bari, Via Ospedale di Venere, 1, 70012 Bari, Italy; 8 Imperial College Ophthalmic Research Group (ICORG) Unit, Imperial College, London NW1 5QH, UK; 9 GENOFTA Srl, Sant’Agnello, 80065 Naples, Italy

**Keywords:** dry eye disease, eye drops, contact lens, hypromellose, 2-methacryloyloxyethyl phosphorylcholine

## Abstract

*Background and Objectives*: Dry eye disease (DED) affects 5–50% of the global population and deeply influences everyday life activities. This study compared the efficacy, tolerability, and safety of novel Respilac artificial tears containing lipidure and hypromellose (HPMC) with the widely used Nextal artificial tears, which are also HPMC-based, for the treatment of moderate DED in contact lenses (CL) wearers. *Materials and Methods*: In a prospective, single-center, randomized investigation, 30 patients aged ≥18 years, diagnosed with moderate DED, and wearing CL were randomly assigned to the Respilac (*n* = 15) or Nextal group (*n* = 15). Patients self-administrated one drop of Respilac or Nextal in both eyes three times daily for 21 days. Changes in the endpoint (visual analogue scale (VAS) score for ocular tolerability, symptom assessment in dry eye (SANDE) score, non-invasive first break-up time (NIF-BUT) results, tear analysis value, meibography results, and CL tolerability results were assessed, comparing treatment groups and time-point evaluations. Adverse events (AEs) were also recorded and evaluated. *Results*: VAS scores decreased with time *(p* < 0.001) in both groups, showing no statistically significant difference among them (*p* = 0.13). Improvements were also detected from screening to end-of-treatment, which were indicated by the SANDE scores for severity and frequency (*p* < 0.001) and by tear analysis results (*p* < 0.001) with no observed difference between the Nextal and Respilac arms. NIF-BUT, meibography, and CL tolerability values were shown to be non-significantly affected by treatment and time. There were no AEs detected in this study cohort. *Conclusions*: Respilac was confirmed to be effective, safe, and well-tolerated. Lipidure-based ophthalmic solution was shown not to be inferior to the currently used Nextal, however, showing improvements in DED symptoms. Within the existing literature, our study is one of the first to report that MPC plus HPMC-containing eye drops are an effective option for the treatment of moderate dry eye disease and desiccation damage prevention in contact lens wearers.

## 1. Introduction

The most common problem associated with contact lens wearing is the development of dry eye disease (DED), which affects approximately 50% of contact lens wearers [1,2,3]. A recent review from the Tear Film & Ocular Surface Society Dry Eye Workshop (TFOS DEWS II) Committee estimated that the prevalence of DED ranged from 5 to 50% of the global population, in both contact lens wearers and non-wearers, showing an increase with age which tends to be greater in females and in Asians [4,5].

Dry eye disease is a multifactorial disorder of the tear film and the interpalpebral ocular surface resulting in ocular discomfort, visual disturbance, and tear film instability. According to the TFOS DEWS II report, DED is accompanied by increased osmolarity of the tear film and inflammation of the ocular surface [4,6]. The main recognized etiological subtypes are tear evaporation, tear hyposecretion, or their combination [4,7].

DED is a chronic or intermittent condition characterized by symptoms of burning, redness, itching, photosensitivity, visual blurring, and foreign body sensations [8,9]. Environmental factors, infections, endogenous stress, exposure to antigens, drugs, and genetics are examples of the main risk factors triggering this disorder [8,9,10]. The prolonged use of computer screens (e.g., computers, tablets, smartphones) and the use of contact lenses can lead to the common symptoms of dry eye disease [9,10,11,12]. In particular, the use of contact lenses can influence the tear evaporation rate, resulting in a greater incidence of DED [2,13].

During the current coronavirus disease-2019 (COVID-19) pandemic, eye dryness and ocular disorder have been increasingly reported in face-mask wearers, leading the scientists from the Centre for Ocular Research & Education (CORE), Waterloo, Canada to define the mask-associated dry eye (MADE) condition [14,15]. Besides increasing DED incidence, MADE condition can also aggravate symptoms in patients with pre-existing dry eye disease [14,16]. Since the use of face masks was made mandatory in many countries to prevent the spread of COVID-19, it has been of fundamental interest to improve dry eye symptoms to avoid discouraging people from wearing face masks. Subjects who are forced to wear masks for a long time (health workers, school workers, students, etc.), however, tend to prefer the use of contact lenses rather than glasses to avoid fogging of the lenses, which forces them to undergo frequent and difficult cleansing.

The management of DED relies on a stepwise approach depending on its severity and cause [8]. The first-line approach includes the removal of aggravating factors (e.g., smoke, prolonged screen viewing) and modification of environmental conditions (e.g., enhanced humidity and less dryness and wind), followed or associated with the application of topical ocular lubricants such as artificial tears, gels, and ointments. When the disease is more severe, further approaches include the administration of topical corticosteroids and anti-inflammatory therapies, topical antibiotics, the systemic use of antioxidants, and surgical punctual occlusion treatment [8,9].

Various groups of drugs are commonly used in the treatment of more severe stages of dry eye disease, including immunomodulatory agents and autologous serum tears. Artificial tears can include ingredients like carboxymethylcellulose, hydroxypropyl methylcellulose, sodium hyaluronate, etc. Lubricant ointments can contain white petrolatum, mineral oil, and lanolin. Cyclosporine A and lifitegrast ophthalmic solution can be considered in severe chronic dry eye disease. Prednisolone acetate, fluorometholone, dexamethasone, and other forms of cortisone are normally added to therapy in acute phases. Autologous serum tears, which are prepared from the patient’s own blood serum, contain growth factors and other natural components that can be used to promote healing in select patients. These medications should be prescribed and supervised by an eye care professional. The choice of treatment depends on the underlying cause and severity of the dry eye symptoms.

Artificial tears, which represent the mainstay for mild-to-moderate DED, are aqueous solutions containing polymers, electrolytes, solutes, and, in some cases, preservatives. The polymers used in dry eye lubricants, due to their viscosity characteristics, can increase tear film stability, reduce ocular surface stress, and improve the optical quality of the surface, thus counteracting eye desiccation [4,17]. Most commercially available solutions contain cellulose derivatives like hydroxypropyl methylcellulose (hypromellose; HPMC) and carboxymethylcellulose (CMC), polyvinyl derivatives, chondroitin sulfate, and sodium hyaluronate [17].

Natural tears contain a complex composition of water, salts, hydrocarbons, proteins, and lipids [18] that artificial tears cannot mimic. This leads to the production of new formulations capable of reproducing the beneficial properties of natural tears.

Respilac (Sooft Italia Spa) is a preservative-free ophthalmic solution, containing lipidure (2.0%) and HPMC (0.1%) as principal components. Lipidure is a novel polymer ingredient with high hygroscopicity and moisture retention capability, consisting of 2-methacryloyloxyethyl phosphorylcholine (MPC). Lipidure has the potential to be an ideal ingredient for artificial tears, considering that it is characterized by a high biological compatibility. This is due to the properties of the phosphorylcholine group, a living organism-derived functional group. Preclinical studies have confirmed the power of lipidure to protect both corneal epithelial cells and contact lenses from drying damage [19,20]. As reported by Ayaki et al., lipidure-containing eye drops are tolerated by the ocular surface cells in the same way as single doses of clinically approved drugs containing sodium hyaluronate. They caused neither severe cytotoxicity nor aberrant cell proliferation. Olivieri et al. [20] demonstrated that the MPC-containing ophthalmic solution was as effective as hyaluronic acid and HPMC 0.3% (Nextal) in providing protection for the ocular surface and desiccation damage prevention in experimental models.

The purpose of this clinical study was to demonstrate the non-inferiority of Respilac eye drops to widely used Nextal artificial tears (containing HPMC 0.3%, N-hydroxymethyl glycinate, and other amino acids; Sooft Italia Spa) in the treatment of moderate DED in contact lens wearers. The study also assessed other potential benefits from this new eye drop in our small cohort of patients.

## 2. Materials and Methods

### 2.1. Study Design

This was a post-marketing, prospective, single-center, randomized single-blind investigation performed at the Ophthalmic Clinic of Santa Marta Hospital, Catania from January 2020 to February 2020. The local Ethics Committee, Comitato Etico Catania 1, approved this investigation on 25 July 2019 (protocol: Resp2018; Rif: 002068). The study was conducted in accordance with the Declaration of Helsinki, good clinical practice, and all applicable regulatory requirements. Patients provided informed consent before undertaking any treatment-related procedures.

### 2.2. Inclusion and Exclusion Criteria

Patients eligible for the study were ≥18 years old and were diagnosed with moderate dry eye disease—grade 1 or 2 according to the DE severity level table proposed by the report of the Diagnostic Methodology Subcommittee of the International Dry Eye Workshop and modified according to Sullivan et al. [21]—for at least 6 months; wore contact lenses; had DED signs and symptoms at least in one eye; and had a best corrected distance visual acuity (BCDVA) ≥ 0.1 decimal units in both eyes at the screening visit. Patients were excluded from the study if they had medium–severe dry eye disease—grade 3 or 4 according to the modified DE severity table; had a BCDVA < 0.1 decimal units in both eyes at the screening visit; had an active ocular infection in both eyes; had a history or presence of ocular surface disorders or eyelid abnormalities unrelated to dry eye disease in both eyes; had eye surgery in both eyes within 90 days before enrollment; presented clinical conditions that could significantly alter the efficacy or evaluation of the medical device under investigation; had hypersensitivity and/or allergy to any of the investigational products; were enrolled in the concomitant trial; were taking ocular products for the treatment of eye disorder; or were taking topical cyclosporine, topical corticosteroids, or any other drugs for the treatment of dry eye in both eyes within 30 days before the screening visit. Female patients who were breastfeeding or pregnant at screening visits or refused to use a highly effective method of contraception were not eligible for the investigation.

In the study, half of the subjects instilled Respilac, while the other half used Nextal drops according to the randomization scheme generated by the randomization.com website. The diagram has been included in the Supplementary Material section at the end of the manuscript.

### 2.3. Objective and Endpoints

The primary objective of the study was to evaluate whether Respilac was inferior to Nextal in terms of safety, tolerability, and efficacy in the treatment of dry eye disease in contact lens wearers. The secondary objectives included confirming the efficacy and safety of the treatment with Respilac.

The endpoints, related to the primary and secondary objectives of the study, were the visual analogue scale (VAS) score for ocular tolerability; symptom assessment in dry eye (SANDE) score (in order to demonstrate that patients had the entire range of possible perceptions of symptoms at their disposal when responding, one end of the scale represents the maximum conceivable symptom strength (i.e., 100%) and the other end represents no symptoms whatsoever); non-invasive first break-up time (NIF-BUT) test (a value in the range of 20–30 swas considered normal, while a value below 10 s was a pathological result; and the tear analysis value, which was assessed by the meniscometry test. The meibography and a graduate scale of glandular tissue loss area were used to study meibomian gland disease (MGD) abnormalities. Endpoints were evaluated according to protocol guidelines at day −3, day 0, day 7, day 14, day 21, and day 24.

#### 2.3.1. Efficacy Assessment

Foreign body sensation, burning/tingling, itching, pain, feeling of sticking, blurred vision, and photophobia were the parameters evaluated using a 100 mm VAS scale for the determination of ocular tolerability. The SANDE questionnaire consisted of a 100 mm horizontal VAS scale to evaluate the frequency and severity of symptoms related to dry eye disease [22,23]. The frequency score evaluation ranged from “rarely” to “all of the time”, while the severity score evaluation ranged from “very mild” to “very severe” [24]. For both VAS questionnaires for ocular tolerability and the SANDE questionnaire, total VAS scores were reported as average scores (higher scores representing greater eye discomfort). NIF-BUT was measured using automated detection of the first break-up, while the patients were asked to keep their eyes open and refrain from blinking. NIF-BUT was evaluated as the mean of three consecutive measures per eye [25]. The tear analysis value was assessed using the meniscometry test, while changes in the meibomian gland were evaluated through image meibography and scored from 1 to 3 (Grade 1: mild; Grade 2: medium; and Grade 3: severe) [25,26,27,28].

#### 2.3.2. Tolerability Assessment

To assess CL tolerability, patients were instructed to record the number and level of discomfort events according to a four-point grading scale (0: normal, 1: mild, 2: moderate, 3: severe) in the ‘Patient Diary’ daily and to return the records to investigators.

#### 2.3.3. Safety Assessment

Patients were instructed to report to investigators any adverse event occurrence during the treatment duration. Adverse events were also evaluated through slit lamp biomicroscopy. For the sake of completeness, the results of the keratoscope analysis, intraocular pressure (IOP) test, and visual acuity test were also evaluated. Visual acuity and IOP were evaluated only at screening and on day 21 [25].

### 2.4. Treatment and Assessment

In this clinical investigation, patients were randomly assigned to Respilac or the control group (Nextal) ophthalmic solution according to a randomization scheme provided by the website randomization.com. The treatment cycle duration was 21 days. Patients were instructed to self-administer one drop of Respilac or Nextal (according to the assigned randomization code) in both eyes three times a day every 4 h ± 1 h. According to the protocol, patients underwent six clinical examinations. Patients underwent: (i) a screening visit (day −3), scheduled three days before treatment initiation to allow the washout period using 3 days of saline-only eye drops; (ii) a baseline visit (day 0), during which patients received their randomization assignment and started the treatment; (iii) a first follow-up visit (day 7) 7 days after baseline ±1; (iv) a second follow-up visit (day 14) 14 days after baseline ±1; (v) a third follow-up visit (end of treatment, day 21) 21 days after baseline ±1; and (vi) a final visit (day 24) 3 days after end of treatment for the washout period. No concomitant medications were allowed during the treatment period. The first author (C.G.) was the only evaluator collecting results in order to limit inter-operator variability. All measurements were taken during the morning clinic routine hours from 9:00–11:00 am. Artificial tears were administered one hour before measurements.

### 2.5. Statistical Analysis

The sample size calculation was based on changes in the clinical classification of dry eye disease according to the DEWS Report 2017 [25]. On this basis, a total number of 30 patients is required for an estimated power of 80% to establish non-inferiority (α = 0.05) with an estimated 10% potential drop-out. The sample size calculation was based on changes in the clinical classification of dry eye disease according to the DEWS Report 2017 [25]. On this basis, a total number of 30 patients is required for an estimated power of 80% to establish non-inferiority (α = 0.05) with an estimated 10% potential drop-out. A preliminary correlation analysis using Pearson’s correlation coefficient was conducted between eyes for VAS, NIF-BUT, tear analysis, and IOP. Variables with a significant correlation greater than 0.70 were analyzed using the average of the right and left eye; otherwise, both eyes were analyzed separately. Continuous variables were described by mean and standard deviation (SD), whereas categorical variables were listed as percentages, both by time and treatment. To investigate primary and secondary objectives, continuous variables were analyzed using a repeated mixed measures model, while categorical variables (meibography and CL tolerability) were assessed with a repeated measures logistic regression analysis, reporting an odds ratio (OR) with 95% confidence intervals (CI) [29,30]. The models included terms for screening assessments, treatment, time, and treatment-by-time interaction. The Studentized maximum modulus method for multiple adjustments was adopted. Outliers detected by the interquartile range (IQR) method were excluded from the analysis. Treatment comparisons between screening and the 21-day time-point were assessed through unpaired t-tests for IOP and Fisher’s exact test for visual acuity analysis. All *p*-values were derived from two-sided statistical tests, considering *p*-values < 0.05 statistically significant. All analyses were performed using SAS ver. 9.4 (SAS Institute Inc., Cary, NC, USA).

## 3. Results

Thirty patients diagnosed with moderate DED wearing contact lenses were enrolled in the study (demographic data shown in Table 1) and treated according to a randomization allocation system (*n* = 15 in the Respilac arm, *n* = 15 in the Nextal arm).

Subjects were followed for approximately one month (21 ± 3 days of treatment, 3 days of washout before, and 3 days of washout after the treatment). All patients completed the entire treatment regimen.

Left and right eyes were significantly correlated for VAS and NIF-BUT, with correlation coefficients of r^2^ = 0.88 (*p* < 0.0001) and r^2^ = 0.78 (*p* < 0.0001), respectively. As the coefficient was greater than 0.70, the VAS and NIF-BUT average values for the right and left eye were analyzed together, whereas the tear analysis and the IOP values for the right and left eyes were analyzed separately as their correlation coefficients were inferior to the fixed limit.

The results of the VAS analysis showed a significant decreasing trend with time (*p* < 0.001) starting from an overall mean (SD) value of 25.0 (7.5) at the screening visit and decreasing to 3.5 (3.1) on day 21. At the screening assessment, mean (SD) values for Nextal and Respilac were 21.3 (5.4) and 28.8 (7.5) (*p* = 0.13), respectively, with similar values and no significant differences at all time points. Overall, there was no significant difference between Nextal and Respilac (*p* = 0.73) (Table 2). A secondary analysis comparing average screening values to the average values detected at the following time points showed a greater VAS reduction in the investigational product arm at all time points, with mean (SD) reductions of −24.2 (9.4) for the Respilac group and −17.1 (6.4) for the Nextal group at day 24, even if this difference was not statistically significant (*p* = 1.00).

**Figure 1:**
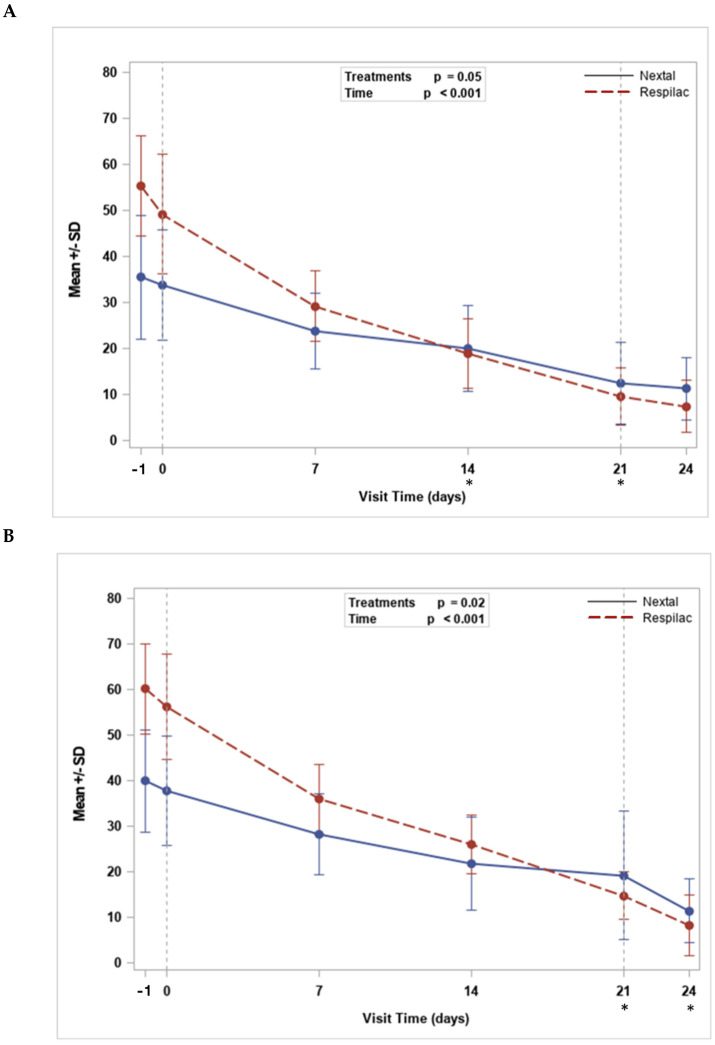
The trend for SANDE severity (mm) (**A**) and SANDE frequency (mm). (**B**) * *p*-value < 0.05 in the comparison between Respilac^®^ and Nextal ophtalmic solutions. SANDE: Symptom assessment questionnaire in dry eye.

The SANDE severity and SANDE frequency scores showed a similar significant decreasing trend with time from screening to day 24 (*p* < 0.001). The mean (SD) values for SANDE severity decreased from 35.5 (13.4) to 11.3 (6.8) and from 55.3 (10.8) to 7.4 (5.7) for the Nextal and Respilac groups (*p* = 0.05), respectively. The mean (SD) value for SANDE frequency reduced from 39.9 (13.4) to 11.4 (6.8) and from 60.1 (9.9) to 8.3 (6.7) for Nextal and Respilac (*p* = 0.02), respectively. Statistically significant differences between the two treatment arms were highlighted at day 14 (*p* = 0.03) and day 21 (*p* = 0.003) for SANDE severity, and a significant treatment difference was highlighted at day 21 (*p* < 0.001) and day 24 (*p* = 0.001) for SANDE frequency (Figure 1).

No statistically significant trend with time was observed in the average values of NIF-BUT (*p* = 0.61). No significant differences between treatment arms were observed at any time points, nor in the overall comparison (*p* = 0.06). Respilac mean values slightly increased from the screening visit to day 21 from a mean (SD) of 8.1 (0.6) up to 12.0 (0.5), while Nextal mean values slightly decreased, starting from a mean (SD) value of 10.8 (6.3) at the screening and decreasing to 8.8 (4.6) at day 21 (Table 3).

**Figure 2:**
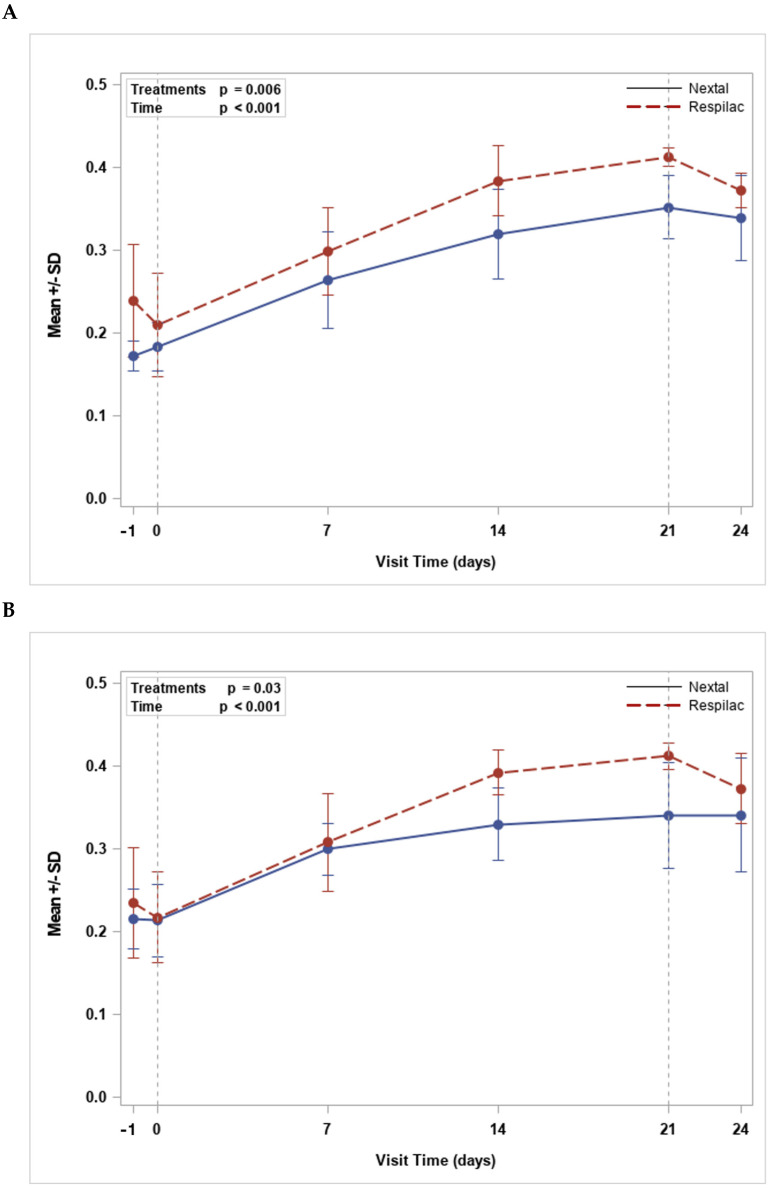
Trend for tear analysis mean values (mm) for right (**A**) and left (**B**) eyes.

Data collected in the tear analysis for both right and left eye evaluations showed a statistically significant increase with time (*p* < 0.001), with no significant difference between treatments for any time-point comparisons. An overall significant treatment difference, however, for both the right eye (*p* = 0.006) and the left eye *(p* = 0.03), was observed. The results showed greater mean values of tear analysis for Respilac when compared with Nextal; however, differences recorded at any assessment time points were not statistically different (Figure 2).

The frequency distribution of meibography scores was not significantly affected by treatment nor by time in the right eye (*p* = 0.41) and the left eye (*p* = 0.34) analysis. Overall, more than 90% of patients, for both right and left eye assessments, had a score of 1 or 2 (mild to medium meibomian gland disease) at any time point. Only one patient (6.7%) assigned to Nextal had a meibography score of 3 upon pre-treatment and at the follow-up. In the Respilac arm, a score of 1 was assigned to 13 (86.7%) patients on days 14, 21, and 24 for both eyes, reaching the highest percentage of patients with a grade 1 meibomian score. Overall, the odds ratios for a score of 2 or 3 vs. 1 were OR = 3.0, 95% CI: (0.2–40.4) and OR = 3.6, 95% CI: (0.3–48.8) in favor of Nextal for the right and left eye, respectively (*p* = not significant). Details are reported in Table 4.

The CL tolerability analysis showed a decreasing trend from day 1 to day 21 in the total number of events per patient, with a discomfort level equal to or superior to grade 1 (*p* = 0.43). In addition, CL tolerability evaluations were not significantly affected by the treatment group (*p* = 0.80) (Table 5).

In the IOP analysis, no statistically significant differences were observed between the two treatment arms for the right and left eye assessments. For the right eye, a decrease in the mean (SD) value of −0.57 (1.22) mmHg was detected from screening to day 21 in the Nextal treatment compared to an increase of 0.13 (2.07) mmHg in Respilac treatment *(p* = 0.28). For the left eye, both treatment arms showed a non-significant decrease from screening to day 21 of −0.86 (1.74) mmHg and −0.07 (2.09) mmHg for Nextal and Respilac, respectively (*p* = 1.00).

Overall, the visual acuity analysis for both left and right eyes detected only three unique values (0.8, 0.9, and 1.0). Right eye visual acuity did not change at 21 days compared to pre-treatment for either Nextal or Respilac while left eye visual acuity did not increase significantly *(p* = 1.00) from 0.9 to 1.0 at 21 days for one patient randomized to Nextal.

No adverse events were reported by patients during the assessment visits. This result was also confirmed by negative values at all the assessment time points and in both treatment groups through keratoscope analysis and slit lamp biomicroscopy.

The study confirms the safety and efficacy of Respilac in dry eye syndrome compared to Nextal based on the results obtained for the visual analogue scale (VAS) score for ocular tolerability, symptom assessment in dry eye (SANDE) score, non-invasive first break-up time (NIF-BUT) result; tear analysis value, meibography result, contact lens (CL) tolerability results, evaluation of reported adverse events, and IOP.

## 4. Discussion

Our study showed that Respilac ophthalmic solution was not inferior to the ophthalmic solution currently used in Nextal in terms of safety, tolerability, and efficacy in the treatment of moderate DED in people wearing contact lenses.

The results of the ocular tolerability VAS analysis showed a significant decreasing trend over time in both groups, with a difference, even if not significant, between Nextal and Respilac. A significant difference, however, was found between Respilac and Nextal when comparing results with pre-treatment conditions, which were in favor of Respilac at all time points. These results confirm the primary outcome of the study, which is that Respilac is not inferior to Nextal in terms of safety, tolerability, and efficacy. In addition, Respilac was shown to have a greater VAS reduction trend compared to Nextal, even if this difference was not statistically significant. Our study is one of the first in the current literature to report that the MPC plus HPMC-containing eye drops are an effective option for the treatment of moderate dry eye disease and desiccation damage prevention in contact lens wearers.

Both SANDE questionnaire analyses showed a decrease in the frequency and severity of dry eye symptoms with time after the treatment with Respilac or Nextal, confirming the efficacy of both solutions in the treatment of DED. Moreover, Respilac seems to lead to better results when compared with Nextal, especially after 14–21 days of treatment. Regarding tear analysis, Respilac was confirmed as a non-inferior treatment to Nextal and as an effective treatment for DED. Although our data showed a “normal” tear meniscus height, ranging between 0.2 and 0.5 mm [28], the present analysis detected a significant increasing trend with time with no significant difference between treatments.

The NIF-BUT results showed a non-significant trend over time for Nextal and for Respilac. The values obtained in both the treatment groups were approximately 10 s, which is the threshold value to diagnose a tear film instability [26,31]. For this reason, it could be assumed that the use of Respilac and Nextal contributed to maintaining a rather constant NIF-BUT value over time. In both randomized arms, meibography scores were not significantly affected by treatment or time. Even in this case, it could be assumed that the use of Respilac and Nextal enabled the enhanced or constant meibography score over time. The tolerability of Respilac was also evaluated utilizing CL tolerability analysis, which showed an overall decreasing trend in the number of events from day 1 to day 21.

Concerning safety, both Respilac and Nextal eye drops presented an excellent safety profile considering that the results obtained through keratoscope analysis were “negative” at all the time points, and in both treatment groups, no adverse events were reported by patients for the entire study duration.

Our study demonstrated, for the first time in a clinical trial, that the novel Respilac ophthalmic solution, containing lipidure, is safe and effective in the treatment of moderate DED in contact lens wearers. The data collected in this investigation showed the value of lipidure in protecting against eye desiccation damage, which is probably due to its hydrating characteristics that have already been reported in preclinical studies [19,20].

Up to now, artificial tears containing HA, HPMC, and CMC have been considered as the medical devices directed to the treatment of DED which provide the greatest comfort results [17,32]. By demonstrating the non-inferiority of Respilac compared to an extensively used HPMC artificial tear (Nextal), it can be assumed that even the MPC plus HPMC-containing eye drops could be considered an effective device for the treatment of moderate dry eye disease.

Both subjective and objective parameters were collected and analyzed. In our study, patient-reported symptoms in VAS and SANDE questionnaires and clinical signs evaluated according to NIF-BUT and meibography showed an overall lack of concordance. These results are in agreement with previously published studies [33,34] reporting no correlation between the signs and symptoms of DED. These observations can be explained by the deep impact of DE symptoms, rather than signs, on the quality of life (QoL) of patients and, especially, on vision-related QoL [35,36]. Foreign body sensation, burning, itching, pain, feeling of sticking, blurred vision, and photophobia represent a limitation in everyday life, especially in vision-related activities like reading, driving, and working at the computer. In addition to clinical examinations, it is also important to assess patients’ symptoms and QoL scores to better assess patients’ responses to treatment.

The limits of the present investigation are represented by the small cohort of patients and by the short-term follow-up. Further studies with a larger sample size are needed to soundly demonstrate the efficacy of Respilac in the treatment of DED. Since dry eye is a chronic or intermittent disease [9], studies with a longer on-treatment duration and post-treatment phase would allow a more accurate examination of the safety profile of this device.

## 5. Conclusions

In conclusion, our study supports the fact that Respilac is effective, safe, and well-tolerated, as well as a currently used HPMC ophthalmic solution, for the treatment of moderate dry eye in subjects that wear contact lenses. The increased viscosity characteristics of MPC and HPMC help to enhance tear film stability, reduce ocular surface stress, and improve the optical quality of the surface, thus contrasting eye desiccation. MPC plus HPMC-containing eye drops can provide an effective option for the treatment of moderate dry eye disease and desiccation damage prevention in contact lens wearers.

## Figures and Tables

**Table 1:** Demographic characteristics.

		Treatment, Mean (SD)		
	All Patients N = 30	Nextal N = 15	Respilac N = 15	*p*-Value
Sex (F/M)		11/15	10/15	*p* > 0.50
Race (%)		Caucasian 100	Caucasian 100	*p* > 0.50
Age (y) (mean ± SD)	26.3 ± 6.52	26.66 ± 6.37	25.93 ± 6.87	*p* > 0.50

**Table 2:** VAS for ocular tolerability average scores for both eyes by time and treatment.

		Treatment, Mean (SD)		
Visit Time (Days)	All Patients N = 30	Nextal N = 15	Respilac N = 15	*p*-Value ^a^
Screening (day −3)	25.0 (7.5)	21.3 (5.4)	28.8 (7.5)	0.13
Day 0	25.4 (7.6)	21.5 (5.2)	29.3 (7.6)	0.07
Day 7	14.4 (4.7)	13.9 (4.2)	14.9 (5.3)	1.00
Day 14	7.1 (3.4)	7.5 (3.2)	6.6 (3.5)	0.78
Day 21	3.5 (3.1)	4.3 (3.2)	2.7 (2.9)	0.36
Day 24	4.4 (2.5)	4.2 (2.7)	4.6 (2.3)	1.00
Overall	13.3 (10.5)	12.1 (8.4)	14.5 (12.2)	0.73

VAS: Visual analog scale. Main effects: treatment, *p* = 0.73; time *p* < 0.001; pre-treatment VAS *p* < 0.001; interaction: treatment × time, *p* < 0.001; ^a^ adjusted for multiple comparisons.

**Table 3:** NIF-BUT average values (seconds) for both eyes by time and treatment.

		Treatment, Mean (SD)		
Visit Time (Days)	All Patients N = 30	Nextal N = 15	Respilac N = 15	*p*-Value ^a^
Screening (day −3)	9.4 (4.6)	10.8 (6.3)	8.1 (0.6)	*1.00*
Day 0	9.2 (4.9)	10.7 (6.7)	7.6 (0.5)	*0.94*
Day 7	9.9 (2.7)	10.0 (3.7)	9.8 (1.1)	*1.00*
Day 14	10.2 (2.2)	9.0 (2.5)	11.3 (0.9)	*0.75*
Day 21	10.4 (3.6)	8.8 (4.6)	12.0 (0.5)	*0.17*
Day 24	9.2 (3.4)	7.7 (4.4)	10.8 (0.5)	*0.23*
Overall	9.7 (3.7)	9.5 (4.9)	9.9 (1.8)	*0.06*

NIF-BUT: Non-invasive film tear break-up time. Main effects: treatment, *p* = 0.06; time *p* = 0.61; pre-treatment NIF-BUT *p* < 0.001; interaction: treatment × time, *p* < 0.001; ^a^ adjusted for multiple comparisons.

**Table 4:** Meibography analysis by time and treatment for right and left eyes.

**Right Eye**		**N (%)**	**Treatment, N (Col %)**	
**Visit**	**Score**	**All Patients** **N = 30**	**Nextal** **N = 15**	**Respilac** **N = 15**
Screening (day −3)	1	18 (60.0)	11 (73.3)	7 (46.7)
	2	11 (36.7)	3 (20.0)	8 (53.3)
	3	1 (3.3)	1 (6.7)	0
Day 0	1	16 (53.3)	8 (53.3)	8 (53.3)
	2	13 (43.3)	6 (40.0)	7 (46.7)
	3	1 (3.3)	1 (6.7)	0
Day 7	1	14 (46.7)	4 (26.7)	10 (66.7)
	2	16 (43.3)	11 (73.3)	5 (33.3)
	3	0	0	0
Day 14	1	19 (63.3)	6 (40.0)	13 (86.7)
	2	11 (36.7)	9 (60.0)	2 (13.3)
	3	0	0	0
Day 21	1	18 (60.0)	5 (33.3)	13 (86.7)
	2	12 (40.0)	10 (66.7)	2 (13.3)
	3	0	0	0
Day 24	1	17 (56.7)	4 (26.7)	13 (86.7)
	2	12 (40.0)	10 (66.7)	2 (13.3)
	3	1 (3.3)	1 (6.7)	0
**Left Eye**		**N (%)**	**Treatment, N (Col %)**	
**Visit**	**Score**	**All Patients** **N = 30**	**Nextal** **N = 15**	**Respilac** **N = 15**
Screening (day −3)	1	14 (46.7)	6 (40.0)	8 (53.3)
	2	16 (43.3)	9 (60.0)	7 (46.7)
	3	0	0	0
Day 0	1	13 (43.3)	6 (40.0)	7 (46.7)
	2	17 (56.7)	9 (60.0)	8 (53.3)
	3	0	0	0
Day 7	1	15 (50.0)	4 (26.7)	11 (73.3)
	2	15 (50.0)	11 (73.3)	4 (26.7)
	3	0	0	0
Day 14	1	18 (60.0)	5 (33.3)	13 (86.7)
	2	10 (33.3)	8 (53.3)	2 (13.3)
	3	2 (6.7)	2 (13.3)	0
Day 21	1	19 (63.3)	6 (40.0)	13 (86.7)
	2	10 (33.3)	8 (53.3)	2 (13.3)
	3	1 (3.3)	1 (6.7)	0
Day 24	1	18 (60.0)	5 (33.3)	13 (86.7)
	2	11 (36.7)	9 (60.0)	2 (13.3)
	3	1 (3.3)	1 (6.7)	0

Right eye. Main effects: treatment, *p* = 0.41; time *p* = 1.00; interaction: treatment × time, *p* = 0.94. Left eye. Main effects: treatment, *p* = 0.34; time *p* = 0.99; interaction: treatment × time, *p* = 0.99.

**Table 5:** Contact lens tolerability: total events (grade ≥ 1) by time and treatment.

		Treatment, Total No. of Events	
Visit Time (Days)	All Patients N = 30	Nextal N = 15	Respilac N = 15
Day 1	14	9	5
Day 7	6	3	3
Day 14	8	5	3
Day 21	4	3	1

Main effects: treatment, *p* = 0.80; time *p* = 0.43; interaction: treatment × time, *p* = 0.92.

## Data Availability

Data are contained within the article.

## Supplementary Materials

The following supporting information can be downloaded at: https://www.mdpi.com/article/10.3390/medicina60020287/s1. The randomization scheme generated from the randomization.com.
