# Supplementary material for: Efficacy and Safety of Artificial Tears Containing Lipidure and Hypromellose for the Treatment of Moderate Dry Eye Disease in Contact Lens Wearers
Source: Medicina (Kaunas). 2024 Feb 8;60(2):287. doi: 10.3390/medicina60020287 (PMC10890511; doi:10.3390/medicina60020287)
Supplement: Supplementary file 1 [file medicina-60-00287-s001.zip › medicina-2828767-supplementary.pdf]

Supplementary material

The randomization scheme generated from the randomization.com

Nello studio proposto, metà dei soggetti riceveranno Respilac®, mentre l'altra metà una lacrima con solo HPMC e aminoacidi (Nextal) secondo lo schema di randomizzazione (dal sito randomization.com) come segue:

1. RespiLAC____________________
2. RespiLAC____________________
3. RespiLAC____________________
4. Next 300____________________
5. Next 300____________________
6. RespiLAC____________________
7. RespiLAC____________________
8. Next 300____________________
9. Next 300____________________
10. RespiLAC____________________
11. Next 300____________________
12. RespiLAC____________________
13. Next 300____________________
14. Next 300____________________
15. Next 300____________________

15. Next 300____________________
16. RespiLAC____________________
17. Next 300____________________
18. Next 300____________________
19. RespiLAC____________________
20. RespiLAC____________________
21. Next 300____________________
22. Next 300____________________
23. Next 300____________________
24. Next 300____________________
25. Next 300____________________
26. RespiLAC____________________
27. RespiLAC____________________
28. RespiLAC____________________
29. RespiLAC____________________
30. RespiLAC____________________
